# Shedding light on dark social: systems-theoretical perspectives on membership-gated digital communication places

**DOI:** 10.3389/fsoc.2026.1872010

**Published:** 2026-08-11

**Authors:** Jakob Wiesinger, Isabel Kusche

**Affiliations:** Institute for Sociology, University of Bamberg, Bamberg, Germany

**Keywords:** closed communication places, digital communication, membership, operational coupling, political communication, structural coupling, systems theory

## Abstract

This article develops a systems-theoretical perspective on digital communication in closed communication places, focusing on membership as the central principle structuring communication in non-public settings. It combines the concept of membership-based social systems with the distinction between operational coupling and structural coupling in order to analyze whether communication in membership-gated digital communication places (MDCPs) may become consequential for the mass media system and the political system. Although MDCPs have become part of everyday digital communication, they remain under-theorized, especially with regard to how they affect the interplay between social systems. The article conceptualizes MDCPs as communicative extensions of membership-based social systems and shows how different membership logics shape the selection and circulation of communication. It argues that increasing operational coupling does not condense into stable structural coupling with the mass media system or the political system. This creates asymmetries of observation: communication in MDCPs may become publicly or politically consequential, while the contexts that make it connectable across social systems remain only episodically observable. MDCPs thus matter because they can irritate the mass media system and the political system without becoming continuously observable to them.

## 1 Introduction

In March 2025, high-ranking US officials used a group chat on the messenger Signal to discuss an upcoming military operation in Yemen. Due to a mix-up, journalist Goldberg (2025) was added to the chat, which led to the unintended publication of its contents. This incident illustrates how closely membership, visibility, and communication flows are intertwined in closed digital communication. What became visible in this case was not only a security failure, but also the social significance of communicative spaces whose boundaries are ordinarily taken for granted and whose internal communication is typically inaccessible to observation.

Research on digital communication has long paid limited attention to such closed communication spaces (Rossini, 2023). Only in recent years has this field received increasing scholarly attention. These spaces are embedded in social media environments, yet the communication taking place within them is neither publicly visible nor searchable. Malhotra (2025) refers to such settings as bounded social media places (BSMPs). Where more than two individuals are involved and access is restricted through membership, we refer to them as membership-gated digital communication places (MDCPs).

The social significance of MDCPs lies in their capacity to circulate content rapidly beyond publicly visible arenas. This has consequences for the dissemination of misinformation (Chadwick et al., 2024), political mobilization (Rheingold, 2003; Ling and Lai, 2016), and the circulation of media content more generally. At the same time, these processes remain difficult to observe. The significance of such circulation has been captured under the term “dark social,” referring to the large share of traffic and dissemination that can be attributed to interpersonal communication (Madrigal, 2012).

Existing research has addressed many important phenomena related to these spaces, but it often remains either problem-centered or methodologically constrained by the limited observability of closed communication. As a result, the boundedness of such spaces is frequently insufficiently theorized. This is particularly problematic because the constitution and maintenance of boundaries are foundational conditions of communicative operations in MDCPs. Against this background, this article develops a systems-theoretical perspective on MDCPs centered on the concept of membership. We draw on approaches that conceptualize social systems between interaction and society—namely family, group, organization, and social movement—to analyze MDCPs as communicative extensions of membership-based social systems (Tyrell, 1983; Kühl, 2015). From this perspective, MDCPs make it possible to observe boundary formation with unusual clarity, since technologically stabilized communication renders otherwise diffuse membership structures empirically more accessible. At the same time, these spaces cannot be understood solely in terms of access restrictions. Their sociological significance lies in the fact that different boundary logics shape different criteria of communicative selection. Building on this, we argue that the circulation of communication in and between MDCPs can be analyzed in two ways. First, communication can be described as operational coupling, that is, as singular communicative events that become connectable across more than one system context without constituting the same operation in each of them. Second, communication in MDCPs can be described as part of structural couplings with the mass media system and the political system. This makes it possible to analyze how the growing relevance—or apparent relevance—of such contexts may alter the self-observation capacities of other social systems. In this sense, however, a growing number of event-based connections does not condense into structures that would stabilize expectations across system contexts.

The article proceeds in four steps. First, it outlines the relevance of bounded social media places (BSMPs), reviews the state of research, and introduces the conceptual shift towards membership-gated digital communication places (MDCPs). Second, communication in MDCPs can be described in relation to structural couplings of the mass media system and the political system. Third, it uses the distinction between operational and structural coupling to examine what communication in MDCPs means for the mass media system and the political system. Finally, it discusses the implications of this perspective for understanding communication, visibility, and self-observation in contemporary digital society, as well as its consequences for further research.

## 2 From bounded social media places to membership-gated digital communication places

Digital communication in BSMPs is socially relevant because it forms part of information cascades that are partially fed by public discourse while simultaneously feeding back into it (Gursky et al., 2022). The horizontal dimension of this circulation also takes place within BSMPs. It is associated with phenomena such as the spread of misinformation (Chadwick et al., 2024), political mobilization (Ling and Yttri, 2002; Rheingold, 2003), and processes of political opinion formation in the sense of a civic culture (Dahlgren, 2005; Livingstone, 2005).

The dissemination of media content in BSMPs is also relevant for the mass media system. First, there are direct connections between audiences and journalism that can shape agenda-setting processes (Goode, 2009). So-called meso news spaces—news-focused chat groups in which participants actively engage in news-related practices—further illustrate this interrelation (Tenenboim and Kligler-Vilenchik, 2020). Moreover, communicative events emerging within these contexts may themselves become objects of mass media coverage (Herbers, 2021; Goldberg, 2025). Beyond these situational linkages, a more structural connection emerges through the circulation of media content within BSMPs. Often described as “dark social,” these forms of distribution are difficult to measure (Madrigal, 2012). However, recent data indicate that applications enabling communication in BSMPs—such as messaging services—are used for news consumption at levels comparable to, or only slightly below, other distribution channels in many Western European countries (Egan et al., 2026). Consequently, the dissemination of media content in these settings is also economically relevant for media organizations, as reflected in the widespread integration of sharing functionalities for messaging services on newspaper websites.

Despite this relevance, existing research on BSMPs exhibits several systematic limitations. Both theoretical and empirical approaches often focus on extreme or exceptional cases such as political radicalization or the spread of misinformation (Gursky et al., 2022; Chadwick et al., 2024). This focus is well justified, but it tends to foreground specific problem contexts. BSMPs, however, are not limited to such contexts; they are embedded in everyday digital media use.

Moreover, empirical research often focuses on chat groups that are publicly accessible, since the object of study implies that the visibility of communication is methodologically difficult and access ethically problematic. Beyond these pragmatic constraints, this tendency indicates that the boundedness of such communication spaces remains insufficiently theorized and is therefore not systematically taken into account when presenting and interpreting empirical findings. As a result, studies frequently refer to closed chat groups while empirically examining publicly accessible formats (Urman and Katz, 2022), and subsequently generalize their findings to closed communication spaces. The constitution and maintenance of boundaries, which distinguish closed from open communication spaces, are thus systematically sidestepped despite their foundational role for the phenomenon under analysis.

Existing research has primarily approached BSMPs through the affordances of digital media, particularly in relation to visibility (Flyverbom et al., 2016; Treem et al., 2020; Malhotra, 2025). While these approaches highlight the enabling role of technology, they provide limited explanations for why specific communicative possibilities are taken up and how participation is effectively restricted. As a result, the social processes through which boundaries are constituted and maintained remain underexplored. One consequence of the limitations identified is that existing research rarely differentiates systematically between different forms of bounded communication. For the present argument, however, such distinctions are crucial, as the selective circulation of communication depends on the specific boundary structures of the contexts in which it occurs. Within BSMPs, a basic distinction can be made between dyadic interactions and constellations of more than two participants. To analyze more stable communicative structures, the latter are of particular interest, as they are more likely to give rise to enduring forms of order (Lindemann, 2010). We therefore define BSMPs with more than two members as membership-gated digital communication places (MDCPs).

## 3 Membership-based social systems and MDCPs

The concept of MDCPs emphasizes the need to treat boundaries as socially structured conditions of communication. We therefore build on the systems-theoretical distinction between four types of membership-based social systems—family, group, social movement, and organization (Tyrell, 2006; Kühl, 2015) to distinguish different types of boundaries in MDCPs. These system types differ systematically in how they define membership and regulate boundaries, that is, in the criteria according to which communicative contributions are treated as relevant or not. This provides the basis for conceptualizing MDCPs as communicative extensions of membership-based social systems. Communicative settings that fall under our concept of MDCPs have already been studied in contexts that correspond to these system types: family WhatsApp groups (Aharony and Gazit, 2016; Taipale and Farinosi, 2018; Malhotra, 2023), small private peer groups (Zhu et al., 2024), organizationally embedded or professional association-based group chats (Wang et al., 2022; Mbada et al., 2023), and activist or protest-related WhatsApp groups (Milan and Barbosa, 2020; Udenze, 2025). These studies provide important context-specific insights, but they do not offer an overarching theoretical perspective on MDCPs as communicative extensions of different membership-based social systems.

The constitutive principle of family systems is biographical or biological membership (Kühl, 2015, p. 73). Families are formed through the establishment of a partnership and the inclusion of shared or individual children. Membership through parenthood cannot be completely terminated, neither by parents nor by children. Partnerships also establish membership; while they can in principle be terminated, they often remain as social points of reference (Kühl, 2015, p. 73). Boundaries are thus not defined by explicit rules, but rather through social attributions and life-history ties (Luhmann, 1990, p. 214). Communication in families is based on the expectation to be addressed as individual personalities with unique identities, i.e., as whole persons. The membership logic is particularistic-diffuse, which means that few are included, but for these few, all topics are expected to be communicable in principle. This renders familial communication intimate and at the same time susceptible to disappointment when the expectation is not fulfilled (Halfmann, 1996, pp. 169–172, 180). Unlike other types of systems, family membership is neither freely chosen nor fully reversible.

Groups as distinctive social systems are based on mutual familiarity (Tyrell, 1983); relationships among their members are characterized by immediacy, diffuseness, and relative durability. The central constitutive principle of groups is the sense of belonging among their members (Neidhardt, 2017). Immediacy and diffuseness point to a specific principle of self-selection, while the relatively enduring member relationships point to a convincing principle of boundary formation (Tyrell, 1983, p. 77). Groups consist of distinct individuals in special, non-interchangeable relationships based on mutual acquaintance between all members. These relationships are so close that the absence of individual members is noticed and discussed (Tyrell, 1983, p. 82). Although a group does not automatically disintegrate when individuals leave or new ones join, its ability to compensate for this is severely limited (Kühl, 2015, p. 72). Although there is no fixed maximum size for groups (Tyrell, 1983, p. 84), there are natural limits to mutual familiarity and personal orientation, which means that groups also limit access to internal communication. These boundaries are relatively stable and are defined by voluntary membership.

The constitutive principle of organizations is formal membership (Luhmann, 2000b). Who is considered a member is clearly determined by the organization’s rules; the organization decides who is allowed to belong and who is not (Kühl, 2015, pp. 70–72). Membership, responsibilities, and behavior are tied to institutionalized roles and purposes. Boundaries are established through decisions regarding admission and expulsion. Membership means submitting to the organization’s rules; non-compliance results in expulsion. Decisions regarding admission and expulsion are central instruments for establishing compliant behavior (Kühl, 2015, p. 70). In MDCPs based on organizational membership, these boundaries must be made visible in day-to-day operations and especially during the group’s formation by reflecting the formal boundaries of the organization and regularly updating or reviewing membership status through formal roles. We see this reflected in new formats as well, such as online platforms focused on dating (Peetz, 2024).

Membership in social movements is, in contrast to organizations, fuzzier. A central element of their system-formation process is protest communication. Values are framed within social movements as absolutes in order to mobilize people (Kusche, 2016, pp. 83–84). Protest is a concatenation of communications that demand certain generalized desirable states, such as a clean environment, and identify opponents who ignore the interest in these desirable states (Japp, 1986). Although it occurs in most social movements, there are also those that do not resort to protest. To focus on protest communication is therefore problematic for determining membership status. Protest is intended to serve mobilization and must be accessible to as many people as possible, which is why it usually takes place in public. Consequently, it is difficult to determine who is a member of a movement (Kühl, 2015, p. 71). Not everyone mobilized to participate in a protest event is deeply involved with a social movement. Movement membership is therefore better understood as a gradated form of involvement than as a clearly formalized status. It ranges from activists to participants and sympathizers (Rucht and Neidhardt, 2001, p. 541). MDCPs are especially relevant for activists and other more strongly involved participants, whereas sympathizers are more likely to encounter movement communication in more open formats. MDCPs thus provide an analytical vantage point on how movement membership is communicatively stabilized where public protest is not the primary form of movement-related activity.

While MDCPs are often associated with communication among strong ties, this captures only part of their possible forms of use. MDCPs constituted in the contexts of families or groups indeed tend to be oriented toward strong ties, and some users limit their use to such contexts. However, MDCPs are also common in organizational contexts, for example among employees, in associations, or in sports clubs. They are similarly relevant in the context of political engagement and social movements, where they can support coordination, mobilization, and the selective circulation of information among more closely involved actors. Usage patterns can therefore range from intimate and strongly person-oriented communication to more purpose-oriented and role-based forms of coordination (Wiesinger, 2026). This variation is crucial because actors differ not only in how many MDCPs they use, but also in the types of membership-based contexts to which these MDCPs belong. As a result, they are endowed with different possibilities for circulating information within and between MDCPs.

## 4 Operational and structural couplings

Social systems differ not only in the way they establish boundaries, but also in the criteria used to determine which kind of information they treat as relevant for further communication. This is also true for MDCPs: “The ability to restrict group membership to intended audiences through visibility management mechanisms allows users to receive content from and send content to specific audiences.” (Malhotra, 2024, p. 8).

The relationship of social systems to their environment is highly selective. A social system remains indifferent to many aspects of its nonsocial and its social environment. It operates independently of whatever may happen concurrently outside its boundaries—except for selected conditions in its environment to which it is sensitive and which it therefore considers in its own operations. System-specific social expectations guide this selective attention. Only against the backdrop of expectations can an event in the environment register as an irritation for a social system to which it then can react in line with its own operational logic. For example, a family system will remain indifferent to a multitude of political, economic, or media events, as long as they do not concern the mutual expectations of family members to be addressed as individual personalities with unique identities; once a family member becomes a passionate member of a political party, though, the system-internal expectation to consider members as whole persons would imply that the planning of family gatherings takes into account whether they conflict with political events, such as campaigning in the run-up to an election. The systems-theoretical concept of structural coupling captures this concurrence of operative closure and selective relations to a social system’s environment (Kusche, 2008, pp. 212–216; Luhmann, 2012, pp. 52–63, Luhmann, 2013, pp. 108–114). This environment encompasses psychic systems as well as other social systems.

Structural couplings are relatively stable relations of selective irritability between systems. Irritations may trigger changes in system structures, but they do not determine them causally (Luhmann, 2012, pp. 54–68, Luhmann, 2013, pp. 108–114). In this sense, interactions between system and environment take the form of “reciprocal perturbations” that can induce structural change without prescribing it (Maturana and Varela, 1987, p. 85). Structural coupling can thus be understood as a stabilized relation in which certain environmental inputs are recurrently made available for system-internal processing (Luhmann, 2012). It is particularly relevant for understanding the relationship between societal function systems. For example, taxes and charges structurally couple the political system and the economy; property and contract structurally couple the legal system and the economy (Luhmann, 2013, pp. 108–113).

In the case of a social system that processes selective irritations from another social system, structural coupling can be supplemented by operational coupling, since both systems employ the same type of operation, namely communication (Schneider, 2009, pp. 353–360). An operational coupling is momentary in the sense that two systems refer to the same communicative event, but each integrates it into its respective concatenated string of communications according to its own logic, i.e., social expectations. Operational couplings are thus singular events in which communication becomes connectable across more than one system context without constituting the same operation in each of them. Each such communicative operation is, as Schneider (2009, p. 104) puts it, Janus-faced: it is an operation in each of the coupled systems and yet not the same operation for all of them.

The observation of such coupled operations always takes place from the perspective of the respective observing system. As Fuchs (1992, p. 183) argues, communicative events coincide in their actuality insofar as they are observed, but not in how or as what they are observed. Rather than a single event directing observation across systems, what emerges is a *multiple event* (*Mehrerlei-Ereignis*): a plurality of event-identities that are constituted simultaneously through the distinctions guiding observation in different systems (Fuchs, 1992, pp. 183–184). For example, the announcement of a new data center project is an event both in the economic system and the political system. In the former system it will likely trigger communications about financial investments as well as increased energy consumption and its consequences for households in the region. In the latter system, it will likely trigger communications expressing support or rejection, typically along the lines of the distinction between government and opposition. Accordingly, if the sharing of information is analyzed from the perspective of concatenating communication, the meaning of an event is defined by the subsequent events that connect to it (Schneider, 2009, p. 104). The same communicative act can therefore generate different concatenations depending on the system context in which it is taken up. It “becomes” an economic event for the economy and a political event for politics as a result of the concatenating communications that refer to it. In contrast, structural coupling means that systems not only occasionally share a communicative event but permanently and mutually rely on aspects of the respective other system as part of their environment.

The technical infrastructure of MDCPs facilitates the multiple character of communicative events. Messages posted in one MDCP can easily be reposted in another. The same applies to social media posts or content from news websites. Media organizations even encourage the posting of links to their news stories in private messengers by providing buttons that automate the necessary steps. MDCPs therefore have the potential to create operational couplings between communicative contexts that did not previously exist.

## 5 Operational coupling via MDCPs

What is true for communication in general also applies to the dissemination of mass media content. It can be observed by other social systems and may trigger irritations there. An observed media report will acquire different meanings depending on the communicative context in which it is processed. The relevance of mass media communication depends on system-specific selection. Each social system processes environmental events according to its own distinctions and only those events that allow for concatenation within the system become relevant.

While communication in MDCPs is processed according to the differing membership logics of families, groups, organizations, and social movements, this logic may include openness to the integration of news content published in legacy media or social media. Such content may be disseminated within as well as between MDCPs and give rise to information cascades, which refer to the environment of MDCPs and yet do so according to their respective logic. Whether a media item is taken up within a family-related MDCP, an organizational context, or a movement-related chat group therefore depends on different criteria of selection. The question is always whether a given event can be rendered communicatively connectable within a particular system. The stability of such selections is secured by system boundaries that realize an inside/outside distinction (Fuchs, 1992, p. 184). For the relationship between MDCPs and the mass media system, this implies that operational coupling occurs whenever communication within an MDCP refers to mass media communication.

Differences in MDCP usage patterns can be interpreted as shaping the conditions under which communicative events become connectable across contexts. For instance, if an actor predominantly uses MDCPs associated with social movements, politically relevant information may become connectable within these contexts and, if taken up, may also be re-articulated in other movement-related MDCPs. In such cases, the selection of communication is oriented toward the goals of the movement and its normative framework.

A different constellation emerges when actors primarily participate in MDCPs related to families or groups. Here, points of connection to politically relevant information may also exist, but whether such content is taken up depends more strongly on its relevance for relationship maintenance, which is based on mutual familiarity (Goh et al., 2019). Operational couplings are therefore less likely to be realized through political relevance alone and instead depend on whether communication can be concatenated with further communication within the respective system.

Consequently, it is highly unlikely that the same media item will circulate uniformly across all MDCPs of which an actor is a member. In many cases, it will not appear as a relevant environmental reference at all. Political information will tend to be integrated into an MDCP’s communication if one of two conditions is fulfilled:

- The MDCP supports the maintenance of specific (in contrast to diffuse) relationships oriented toward shared purposes or interests and the respective political information is relevant to these purposes or interests. This condition is most likely fulfilled in MDCPs embedded in organizations, project-based contexts, leisure activities, or social movements, where communication is structured by functional or normative orientations.
- The MDCP supports the maintenance of diffuse, intimate relationships, typical for families or close friendship groups. Here, the overarching norm of openness to all personal facets of members invites the articulation of a wide range of concerns. The sharing of news items may occur as expressive communication, for instance when such items are considered relevant to particular members or to the relationship itself.

## 6 The mass media system and the question of structural coupling

According to conventional systems-theoretical wisdom, operational couplings cannot replace structural couplings but presuppose them. They condense and actualize mutual irritations between systems and thus enable more immediate and finely tuned information processing (Luhmann, 1998, p. 788). The economic system, the scientific system, but most prominently the political system, are established examples of social systems structurally coupled with the system of mass media.

Do MDCPs need to be integrated into an understanding of structural couplings with the system of mass media? No MDCP is permanently dependent on irritation from mass media communication. Some MDCPs may permanently rely on news reports to inform discussion and planning of courses of action, but the most likely reason is that they are embedded in the context of a social movement or a political organization. The relevant structural coupling is therefore the one between the political system and the mass media system.

However, MDCPs are clearly regarded as relevant communication places within the mass media system. They are treated and reflected as important channels for the dissemination of journalistic content. A significant share of media circulation takes place through interpersonal communication in messaging settings, often described as “dark social” (Madrigal, 2012). From the perspective of media organizations, these forms of dissemination are not directly observable and can only be inferred indirectly, yet they are of considerable economic relevance, as they contribute to audience reach and are actively supported through sharing functionalities.

In this respect, they resemble the role of social media from the point of view of journalism. Boccia Artieri and Gemini (2019) use the notion of structural coupling to make sense of the relationship between the system of mass media and social media platforms or the web more generally. They understand both as media of dissemination, but highlight that they do not merge into a new, hybrid media system (Chadwick, 2017), but maintain distinct boundaries. Mass media create the same experience for many, while social media, due to the algorithms they employ to shape what users can see, create different experiences for each user (Boccia Artieri and Gemini, 2019, p. 8). In other words, mass media offer the same opportunities for concatenating communication in many contexts simultaneously whereas social media offer opportunities for concatenating communication pre-selected for the context of a particular user. Consequently, social media can be conceived as a new, additional environment of the mass media system (Boccia Artieri and Gemini, 2019).

Trying to apply the same reasoning to MDCPs runs into theoretical difficulties. Firstly, while the selectivity of social media is at least partially based on algorithms and personalization, information in MDCPs is not pre-selected by algorithms and there is not even a general preference for information. Mass media employ the code information/non-information, assuming a generalized audience and social media sites distinguish between information and non-information for each single user (Boccia Artieri and Gemini, 2019, p. 9). In contrast, the extent to which communication in MDCPs follows a preference for information varies substantially.

**Figure 1:**
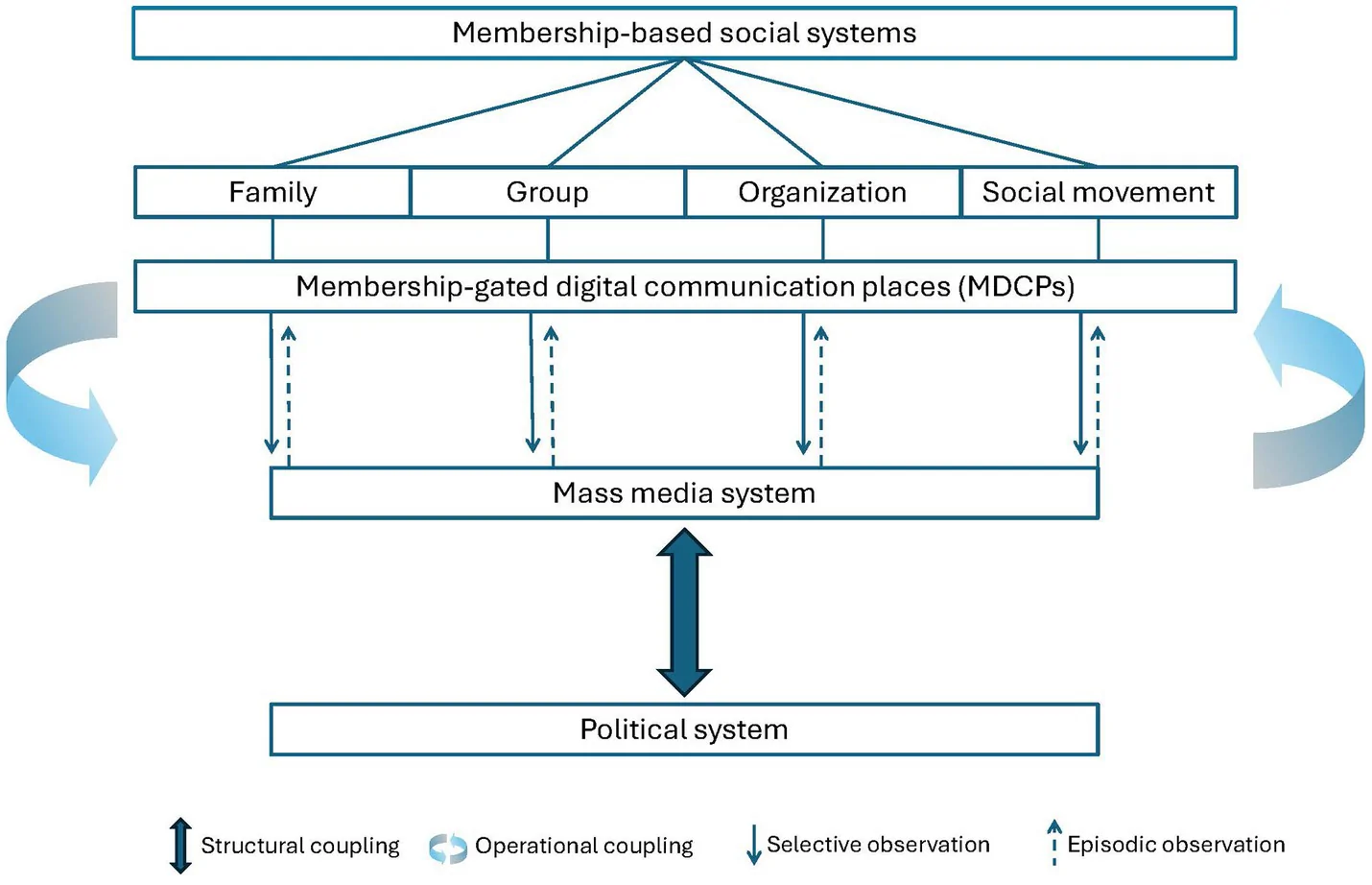
Operational and structural coupling in membership-gated digital communication places (MDCPs). The figure illustrates how MDCPs are connected to membership-based social systems and how communication in MDCPs may give rise to operational couplings with the mass media system and the political system without condensing into stable structural couplings. It also indicates the asymmetry of observation: while communication in MDCPs may become publicly or politically consequential, the communicative contexts in which such connections emerge remain only episodically observable.

Secondly, some communications on social media gain high visibility because algorithms amplify their reach and may even become a topic of mass media coverage, for example via reporting on hashtags or trending topics (Boccia Artieri and Gemini, 2019, p. 11). In contrast, the mass media system cannot observe what happens to information that originated within it but is then shared in MDCPs. In other words, there is a pronounced asymmetry of observation. While communication in many, though not all, MDCPs refers to content provided by mass media and thus continuously monitors it, the reverse is not the case. Communication within MDCPs remains largely inaccessible to the mass media system and is often only registered indirectly, for instance through aggregated usage data, specific incidents, as well as through the analysis of selected communicative settings such as meso news spaces (Tenenboim and Kligler-Vilenchik, 2020). Figure 1 illustrates the asymmetries of observation and summarizes the role that structural and operational couplings play for it.

This asymmetry also becomes evident when entire communicative communities rely on MDCPs as primary arenas of exchange. As a result, certain topics are predominantly processed within these contexts and do not necessarily enter publicly visible arenas. Such dynamics have been described, for example, in studies of diaspora communities, where communication within closed networks plays a central role in shaping interpretations of public events while remaining only partially translated into publicly observable discourse (Trauthig and Woolley, 2023). This can lead to structural absences in mass media communication, as topics to which the code information/non-information would be sensitive in principle remain confined to specific communicative contexts.

The mobilizing potential of such less publicly observable communication has already been anticipated in earlier accounts of mobile communication (Ling and Yttri, 2002; Rheingold, 2003). Contemporary messaging settings extend this logic. Empirically well-documented European cases do not always concern MDCPs in the narrow sense, since available data often come from Telegram channels and large group chats rather than from fully closed interpersonal groups. Nevertheless, they show how messaging-based settings can function as arenas of mobilization whose communicative dynamics are only partially observable from the perspective of the mass media system. The German Corona protest movement, especially the Querdenken network, is a relevant case in point: studies of Telegram communication in this context show that movement-related channels were used for inward-oriented communication, message circulation, and calls for online and offline action (Jost and Dogruel, 2023; Zehring and Domahidi, 2023). The resulting protests became visible to mass media as demonstrations, conflicts over pandemic measures, and political radicalization, whereas the communicative processes through which mobilization was prepared, reinforced, and circulated remained only partially accessible. Such cases mark the empirically more accessible edge of a broader problem: if mobilization through public or semi-public messaging settings already creates asymmetries of observation, this problem is likely to be intensified in MDCPs whose membership boundaries are more restrictive and whose communicative pathways are less accessible from the outside.

Taken together, these findings indicate that regular operational couplings do not coincide with a structural coupling between the mass media system and MDCPs. Media content is recurrently made available for selection, while its further dissemination, contextualization, and possible translation into collective action take place in communicative settings that are not directly observable but can only be inferred indirectly. Mobilization events make this asymmetry especially visible: the mass media system can observe demonstrations, blockades, or political conflicts, but it usually observes only retrospectively and incompletely the communicative processes through which such events were prepared, coordinated, or sustained.

## 7 Implications for the political system

The mass media system draws on MDCPs as dissemination media and thus facilitates operational couplings. These operational couplings are more likely with, but not exclusive to, MDCPs whose membership logic follows membership-based social systems that are already structurally coupled to the mass media. The most relevant case is that of systems whose operations are primarily oriented toward the political system.

Yet, the implications of MDCPs for the political system are not limited to this subset of MDCPs. MDCPs in general are increasingly seen as communicative contexts relevant for political influence, mobilization, or the formation of political orientations, and that can be strategically addressed, for example through practices of microtargeting (Evangelista and Bruno, 2019). Such attempts presuppose that communication within MDCPs constitutes a setting in which political messages may be selectively taken up and processed.

Again, there is an asymmetry in observation, although it is less pronounced than in the relation to mass media. Expectations that political communication can be effectively directed into such contexts coexist with limited knowledge about how communication is actually processed there and whether it becomes politically relevant at all (Kusche, 2022). In this sense, MDCPs appear simultaneously as politically promising and politically opaque environments.

In this regard, the relation between MDCPs and the political system mirrors descriptions of the role that civic culture plays in politics. The respective strand of literature notes, long before the spread of MDCPs, that news and political information may be encountered online, yet they are often discussed in interpersonal contexts that are not fully visible to the public sphere (Dahlgren, 2005; Livingstone, 2005; Heikkilä and Ahva, 2014). Not all networks relevant to political opinion formation are digital, and not all communication that later matters politically is directly observable on public platforms. Nevertheless, such communication may reappear in aggregated and publicly visible forms, for example in opinion polls or patterns of political behavior. In some sense, the existence of MDCPs renders the political relevance of various communication networks to political opinion formation more observable than before, but at the same time denies the political system the possibility to observe them reliably.

This selective visibility is not merely a static distortion of publicly observable social media communication. It can also emerge dynamically, as users learn from previous experiences that political expression in visible platform spaces may entail social costs. Users with moderate political positions may withdraw from political expression after experiencing hostility, reputational risks, or a lack of social reward (Bail, 2021). Related empirical work supports this interpretation. Fear of social isolation can foster willingness to self-censor and, in turn, discourage political expression while encouraging withdrawal behaviors on social media (Chen, 2018). Similarly, fear of social sanctions predicts lower levels of political expression on Facebook (Weeks et al., 2023). This pattern can be described as a “self-censoring majority,” in which moderate conservatives, independents, and moderate liberals are more likely to remain silent in the face of highly vocal ideological minorities (Burnett et al., 2022). Publicly visible social media discourse may therefore become increasingly unrepresentative over time: what remains observable is not simply political opinion as such, but the communication of those actors who are willing and able to accept the social risks of public political expression.

This has important implications for the political system’s capacity for self-observation. If less confrontational or more moderate political orientations become less visible in public or semi-public platform spaces, their absence cannot be interpreted as political irrelevance. Political evaluations, acts of circulation, and attempts at orientation may instead shift into less visible communicative settings. MDCPs are therefore relevant not because they replace public political communication, but because they provide socially embedded contexts in which political communication can continue under conditions of restricted visibility and audience-specific addressability.

In this sense, the period in which social media appeared to make a relatively broad range of political debate publicly observable may have been historically brief. However, the erosion of this visibility does not amount to a return to a pre-platform condition of interpersonal political communication. MDCPs transform interpersonal communication by making it technically easier to circulate political content across otherwise distinct membership-based contexts. A link, image, video, or political interpretation can move from one MDCP to another without requiring public posting, while remaining embedded in relations of trust, familiarity, organizational affiliation, or shared political commitment. For the political system, this creates a specific observational problem: politically relevant communication may continue to circulate widely, but it does so through settings whose boundaries, audiences, and connective pathways are only partially visible from the outside.

A political system that operates based on the expectation that MDCPs form part of its environment can react in very different ways. It can accept them as a new source of political uncertainty and continue to rely on the established structural coupling with the mass media system. Yet it can also attempt to control them by ensuring as many operational couplings as possible with MDCPs, for example by using people with many MDCPs as nano-influencers (Woolley, 2022). Since operational couplings in MDCPs depend on their respective members and their MDCPs, these attempts will tend to resemble clientelistic brokering, with influential MDCP users acting as intermediaries between politicians and an audience to which these politicians do not have direct access (Kusche, 2020).

The coupling that is realized in such a way does not mean that communication within MDCPs is directly controllable or transparently observable. Rather, it means that these contexts can be made part of an anticipated environment in which political communication may become relevant, be selectively processed, and contribute to broader political dynamics without being publicly accessible in its concrete form. As these communicative settings gain—or appear to gain—relevance, this also constrains the political system’s capacity for self-observation, since potentially consequential communication remains only indirectly accessible and cannot be fully observed within publicly available communication (Kusche, 2022).

## 8 Discussion and conclusion

The article set out to develop a systems-theoretical perspective on membership-gated digital communication places (MDCPs) in order to better understand how communication becomes connectable within and between closed digital settings and how such processes relate to broader societal dynamics. The opening example of Signal-Gate illustrated this problem in a particularly visible form: communication that was ordinarily membership-gated became publicly consequential only through an exceptional breach of access. It therefore exemplifies an episodic form of visibility in which MDCPs become observable not as continuous communicative contexts, but through singular incidents that render otherwise inaccessible communication available for public and political follow-up communication. By conceptualizing connections in terms of operational and structural coupling, the analysis links singular communicative events to more stable relations of selective irritability between social systems. This makes it possible to specify how communication in MDCPs relates to the ongoing self-reproduction and self-observation of function systems such as mass media and politics, even though the underlying processes remain largely inaccessible to observation across MDCPs.

At the same time, the article advances a methodological argument grounded in systems theory: the analysis of communication cannot rely on the observation of isolated acts of sharing content but must reconstruct the system-specific contexts in which communication becomes—or fails to become—connectable. This requires taking seriously that actors do not participate in communication as unified individuals, but as context-specific positions distributed across multiple membership relations. The circulation of communication depends on the interplay between system-specific selection criteria and the distribution of memberships across contexts. Non-circulation is therefore not simply the absence of dissemination but a theoretically significant outcome that requires explanation. Communication may fail to become connectable not because it lacks intrinsic relevance, but because it does not meet the selection criteria operative within specific systems and therefore offers no point of connection for subsequent communication.

The distinction between operational and structural coupling highlights a central implication of the analysis. The increasing number of communicative connections across MDCPs is unlikely to produce more durable relations between social systems. Operational coupling between social systems is facilitated by the technical infrastructure underlying both websites on the open internet and MDCPs. Yet asymmetric opportunities for observation prevent these event-based connections from condensing into corresponding structural couplings. While mass media can register that communication circulates—for example through viral effects, leaks, or “dark social”—it lacks access to the contexts in which communication becomes connectable. Events therefore tend to appear as contingent surprises rather than as structured and anticipated patterns of irritation.

One consequence is an episodic form of visibility. Topics may emerge abruptly and disappear again without stabilizing into durable trajectories. In temporal terms, communication thus remains tied to singular connective events, that is, operational couplings, rather than developing into sustained sequences of subsequent communication that establish structures of expectation. The resulting dynamics are characterized by short-lived bursts of attention rather than by patterns of relevance sufficiently enduring to support the attention-directing function of public opinion (Luhmann, 1971). This may impact the political system’s capacity to narrow down political issues that appear at its periphery and transform them into specific choices for decision-making in the center (Luhmann, 2000a, pp. 245–247, 316–318).

The analysis has important implications for empirical research. Existing studies of encrypted messaging apps and their political relevance often rely on observable instances of communication, particularly in publicly accessible settings (Rossini, 2023). They also frequently focus on problematic content such as misinformation or politically contentious communication (e.g., Urman and Katz, 2022; Chadwick et al., 2024). Yet misinformation is itself a difficult entry point for studying MDCPs, since the status of a message as misinformation cannot usually be inferred from the act of sharing alone. It depends on an external assessment of its truth status and therefore presupposes an observational position that is difficult to establish in membership-gated communication. Moreover, the shared context that makes a message connectable within an MDCP may also make it unlikely that participants identify it as misinformation. Messages circulate precisely because they fit context-specific expectations, values, or assumptions. Self-reports are therefore of limited help, since actors who circulate such messages typically do not treat them as misinformation. From the systems-theoretical perspective developed here, empirical research should therefore place greater emphasis on reconstructing communicative contexts rather than focusing solely on isolated acts of sharing or on individual actors considered in isolation. Since actors participate in multiple communicative contexts, analyzing single individuals without reconstructing the distribution of their memberships across different MDCPs risks misattributing communicative dynamics that are in fact structured by relations between contexts.

At the theoretical level, the analysis contributes to ongoing debates about visibility and self-observation in digital society. Following Luhmann (1990, pp. 170–182; 2000, pp. 286–298), systems theory has conventionally understood the relationship between the political system and mass media as a structural coupling that provides politics with a medium for second-order observation or, in other words, political self-observation. While social media arguably increased visibility of political communication and thus opportunities for second-order observation, MDCPs point to a different development: potentially consequential political communication can circulate without becoming available to such observation. As communication increasingly takes place in membership-gated contexts, relevant communicative processes remain structurally inaccessible and can only be inferred indirectly. This limits the capacity of systems such as mass media and politics to observe and interpret ongoing communication processes. Although mass media still exists as a medium for political self-observation, the continuous character of such second-order observation is thus potentially undermined. This could facilitate political communication that strategically avoids exposure to critical reactions by political opponents. As a result, the growing—or perceived—relevance of MDCPs may transform the conditions under which the political system, and similarly other social systems, observe themselves, as potentially consequential communication increasingly escapes direct observation.

## Data Availability

The original contributions presented in the study are included in the article, further inquiries can be directed to the corresponding author.
